# A Hyperspectral Bidirectional Reflectance Model for Land Surface

**DOI:** 10.3390/s20164456

**Published:** 2020-08-10

**Authors:** Qiguang Yang, Xu Liu, Wan Wu

**Affiliations:** 1 Science Systems and Applications, Inc. (SSAI), Hampton, VA 23666, USA; Qiguang.Yang@nasa.gov (Q.Y.); Wan.Wu@nasa.gov (W.W.); 2 NASA Langley Research Center, Hampton, VA 23681, USA

**Keywords:** hyperspectral bidirectional reflectance model, BRDF, Ross-Li model, land surface, remote sensing

## Abstract

A hyperspectral bidirectional reflectance (HSBR) model for land surface has been developed in this work. The HSBR model includes a very diverse land surface bidirectional reflectance distribution function (BRDF) database with ~40,000 spectra. The BRDF database is saved as Ross-Li parameters, which can generate hyperspectral reflectance spectra at different sensor and solar observation geometries. The HSBR model also provides an improved method for generating hyperspectral surface reflectance using multiband satellite measurements. It is shown that the land surface reflective spectrum can be easily simulated using BRDF parameters or reflectance at few preselected wavelengths. The HSBR model is validated using the U.S. Geological Survey (USGS) vegetation database and the AVIRIS reflectance product. The simulated reflective spectra fit the measurements very well with standard deviations normally smaller than 0.01 in the unit of reflectivity. The HSBR model could be used to significantly improve the quality of the reflectance products of satellite and airborne sensors. It also plays important role for intercalibration among space-based instruments and other land surface related applications.

## 1. Introduction

Most land surfaces reflect incident radiance anisotropically. The anisotropic properties can be described by the so-called bidirectional reflectance distribution function (BRDF) of the surface. The directional reflective properties of the Earth’s surface are very important for many applications of remote sensing from satellite or airborne platforms. These applications include monitoring of climate changes [1,2,3,4,5], mapping land covers [6,7,8], analyzing vegetation densities [9,10,11], and completing the inter-calibration between different satellite instruments [12,13]. Taking into account of BRDF effects and making proper BRDF corrections are very important for supervised vicarious calibration (SVC) methodology of hyperspectral sensor [14] and for wide-field-of-view optical scanners [15].

The land surface BRDFs have been investigated for decades in both laboratory and field environments. Many empirical, semi-empirical, and physical BRDF models have been developed for homogeneous materials [16,17,18,19,20,21,22,23,24,25]. However, the reflective spectrum of a material obtained in laboratory may not represent the BRDF of the same type of material in the field very well. In addition to different observation geometries for satellite observations, the surface being measured in the field may be covered by dust or its optical properties may be changed by weathering [26]. Many of the land surfaces may not simply consist of a single material. They may consist of various types of bare soils. They also may be partially or completely covered by different grasses, vegetation, trees, snows, ice, or man-made roads and buildings. This complex nature of the Earth’s surfaces makes it very difficult to model the reflection of the realistic land surfaces. In the past 30 years, scientists developed empirical models [27], semi-empirical models [28,29,30,31,32,33,34], and physical models [35] to describe the land surface bidirectional reflectance. To calculate the BRDFs using these models, one has to have several measured parameters available, such as the single scattering albedo or surface Lambertian reflectance, Henyey-Greenstein asymmetry factor, surface anisotropy parameter, and scattering kernels, etc. Usually, this information is not available at hyperspectral resolution over a large range of wavelengths. It is a challenge task to calculate the hyperspectral reflectance based on a BRDF model.

On the other hand, the bidirectional reflective spectra of various land surfaces have been measured in the last few decades. The U.S. Geological Survey (USGS) Spectral Library Version 7 has thousands of spectra measured in laboratories, fields, and airborne experiments [36]. Different optical geometries were used in these measurements. The biconical reflectance factor (BCRF) or bihemispherical reflectance (BHR) method was used in their laboratory measurements to obtain the surface reflectances of various minerals, plants, chemical compounds, and man-made materials. The hemispherical-conical reflectance factors (HCRF) of some land surfaces, including rocks, soils, and natural mixtures of minerals, were measured in the field settings. The HCRF method was also used to measure the reflectance spectra of forested vegetation plots using airborne instruments. Depending on the spectrometer used, the USGS reflectance spectra cover various spectral ranges from 0.2 μm to 3 μm, 0.35 μm to 2.5 μm, 0.37 μm to 2.5 μm, and 1.12 μm to 216 μm, respectively. However, some of the spectra in the library do not include spectral regions where atmospheric water absorbs.

The ASTER Spectral Library in Jet Propulsion Laboratory contains over 2000 measured spectra [37]. The library includes spectra of soils, rocks, vegetations, minerals, water, snow, ice and man-made materials. Some of the spectra cover a range from 0.3 μm to 14 μm or 0.4 μm to 14 μm. These spectra were measured using BCRF geometry for meteorites and some minerals, and directional hemispherical reflectance (DHR) geometry for all other samples.

The Scanning Imaging Absorption Spectrometer for Atmospheric Chartography (SCIAMACHY) sensor measured the Earth’s reflected spectral radiance from 0.24 μm to 2.38 μm with a resolution from 0.2 nm to 1.5 nm [38]. The instrument was launched on board ENVISAT which was operational from March 2002 to April 2012.

The Airborne Visible Infrared Imaging Spectrometer (AVIRIS) is a unique optical instrument that delivers calibrated images of the upwelling spectral radiance in 224 contiguous spectral channels with wavelengths from 0.4 μm to 2.5 μm [39]. The AVIRIS Level 2 data products contain atmospherically corrected surface reflectance spectra for many airborne flight measurements from 2013 to current. The huge AVIRIS database provides great opportunity to validate the land surface BRDF models.

In addition to the above-mentioned Hyperspectral Reflectance (HSR) measurements, there are multispectral and broad band satellite sensors capable of measuring the surface albedos. For example, the Moderate Resolution Imaging Spectroradiometer (MODIS) BRDF/Albedo product provides Ross-Li BRDF parameters at seven narrow spectral bands and the Clouds and the Earth’s Radiant Energy System (CERES) delivers broadband albedo [40,41].

In principal, the HSR, the Multispectral Reflectance (MSR), and the Broad Band Reflectance (BBR) must be highly correlated if they measure the same surface of the earth. Studies show that the monthly mean of the HSRs from SCIAMACHY, the MSRs from MODIS, and the BBRs from CERES are highly correlated over a large latitude region [42]. The MSR data from several multispectral narrow band sensors were actually used to predict the BBRs for the land surface [43]. It is reported that the hyperspectral BRDF of beach sands has weak wavelength dependence [44]. Its spectral features can be captured with only three broad wavelength regions instead of hundreds of individual wavelengths. The principal component analysis (PCA) regression method has been used to construct the hyperspectral land surface reflectance using MODIS surface reflectances [45,46]. However, in works using this method, the maximum number of the principal components (PCs) allowed was limited by the total number of satellite available channels, which is seven for MODIS. The optimal number of PCs was found to be six, and a larger number of PCs would make their results unstable [45,46]. Zoogman et al. [47] applied the same regression approach to the spectral range from 400 to 900 nm with an additional high-resolution vegetation database included in the study. In general, one may expect that more accurate results will be obtained if a larger number of PCs are used. The unstable results are originated from the way the PC coefficients are calculated. We have derived an improved PCA regression algorithm that produces more accurate and robust results as compared to the method described in [45,46,47]. The improved HSBR model developed in this paper enables the use of any number of PCs in the regression process with stable results.

In this work, we develop a Hyperspectral Bidirectional Reflectance (HSBR) model for remote sensing applications. The HSBR model includes two parts: The first part is a very diverse land surface BRDF database with ~40,000 spectra. A principal component analysis is done with this database. Usually 23 leading PCs are enough to represent various land surfaces accurately. The BRDF database is also saved as Ross-Li parameters, making it very easy to be used in popular radiative transfer models, such as MODTRAN. The second part of the model is providing an improved method for generating hyperspectral surface reflectance using multiband satellite measurements. The HSBR model can be easily used to simulate the hyperspectral reflective spectrum for any land surfaces in the wavelength range of 0.2 μm to 5 μm. The HSBR model was validated by comparing the simulated HSRs with the USGS and AVIRIS measured reflectance data for the various land surfaces. The small errors in the simulated reflectances indicate that the HSBR model can be used for intercalibration between different satellite sensors [12,13]; reconstructing lost information in bad spectral bands of a hyperspectral sensor; and bridging the HSR, MSR, and BBR data so that one may use the MSR data to simulate the HSR data for many remote sensing applications.

The BRDF describes the intrinsic reflective properties of the land surface. However, this parameter cannot be measured directly [48]. The measured parameters are the various reflectances and reflectance factors [49]. Though these parameters have different values at the same wavelength, they do have the same wavelength dependence as reflectance and reflectance factor are integrations of BRDF over corresponding finite solid angles. Therefore, the model we developed in this work can be used for BRDF, reflectance, and reflectance factor.

## 2. Simulation of BRDFs Using Ross-Li Model

To demonstrate the proposed HSBR model, we simulated ~40,000 BRDF spectra for various land surfaces. It is well known that the reflective properties of land surface are very hard to obtain due to the complicated surface structures. The geometric structure of the land surface greatly influences its reflectance due to shadowing and multiple scattering [31]. Many bare soils and canopies have strong back scatterings [50,51]. The reflectance decreases when the sensor moves away from the solar direction. This is caused by the increased geometry structure-induced shadows as well as the reduced solar irradiance on the viewed facets in off solar directions [31]. This angle-dependent scattering component is called geometric scattering. Another structure-related scattering is called volumetric scattering. It usually consists of multiple reflections from different components within a volume and produces a minimum reflectance near nadir viewing. This minimum is due to the absorption and scattering of radiation when it transmits from one layer/medium to another in the nadir direction. Scattering by trees, branches, soil layers, and snow layers are typical volumetric scattering. Geometric and volumetric scatterings are important processes in the interpretation of the land surface BRDF.

### 2.1. Ross-Li Model

The semi-empirical Ross-Li model is used in MODIS BRDF/Albedo product. It assumes the land surface reflectance is a sum of three components: Isotropic scattering, geometric scattering, and volumetric scattering [32].

(1) $$B\left(\theta_{i},\theta_{r},\Delta \varphi \right)=P_{1}+P_{2}K_{geo}\left(\theta_{i},\theta_{r},\Delta \varphi ,P_{4},P_{5}\right)+P_{3}K_{vol}\left(\theta_{i},\theta_{r},\Delta \varphi \right)$$

with the Li-Sparse-Reciprocal geometric scattering kernel *K_geo_* given by

(2) $$K_{geo}=\frac{1+sec\theta_{r}^{\prime }sec\theta_{i}^{\prime }+tan\theta_{r}^{\prime }tan\theta_{i}^{\prime }cos\Delta \varphi }{2}+\left(\frac{t-sintcost}{\pi }-1\right)\left(sec\theta_{r}^{\prime }+sec\theta_{i}^{\prime }\right)$$

The Ross-Thick volume scattering kernel *K_vol_* is given by

(3) $$K_{vol}=\frac{\left(\frac{\pi }{2}-\varphi \right)cos\varphi +sin\varphi }{cos\theta_{r}^{\prime }+cos\theta_{i}^{\prime }}-\frac{\pi }{4}$$

The relative azimuth angle is

(4) $$\Delta \varphi =\varphi_{r}-\varphi_{i}$$

and the cosine of the scattering angle is

(5) $$cos\varphi =cos\theta_{r}cos\theta_{i}+sin\theta_{r}sin\theta_{i}cos\Delta \varphi$$

where $\theta_{r}$ is the satellite view zenith angle and $\theta_{i}$ is the solar zenith angle. The other parameters are

(6) $$cos^{2}t={\left(\frac{P_{4}}{sec\theta_{r}^{\prime }+sec\theta_{i}^{\prime }}\right)}^{2}\left[G{\left(\theta_{r}^{\prime },\theta_{i}^{\prime },\Delta \varphi \right)}^{2}+{\left(tan\theta_{r}^{\prime }tan\theta_{i}^{\prime }sin\Delta \varphi \right)}^{2}\right]$$

(7) $$G\left(\theta_{r}^{\prime },\theta_{i}^{\prime },\Delta \varphi \right)=\sqrt{tan^{2}\theta_{r}^{\prime }+tan^{2}\theta_{i}^{\prime }-2tan\theta_{r}^{\prime }tan\theta_{i}^{\prime }cos\Delta \varphi }$$

(8) $$tan\theta_{r}^{\prime }=P_{5}tan\theta_{r}$$

(9) $$tan\theta_{i}^{\prime }=P_{5}tan\theta_{i}$$

Parameter *P*_1_ is the Lambertian scattering component and equal to the bidirectional reflectance for *θ_r_* = 0 and *θ_i_* = 0. It is given by

(10) $$P_{1}=\alpha C+\left(1-\alpha \right)\left[\frac{s}{3}+\left(R_{0}-\frac{s}{3}\right)e^{-LAI\;Q}\right]$$

where α is the area proportion of the land cover with geometric scattering, C is the reflectance of the sunlit crown (geometric scattering part), s represents the Lambertian reflectance of the scattering facets, $R_{0}$ is the Lambertian reflectance of the flat horizontal surface, LAI is the leaf area index, and $Q=\frac{1}{2}\left(sec\theta_{i}+sec\theta_{r}\right)$, which is usually approximated to be 1.5.

Parameter *P*_2_ is the coefficient of the Li-Sparse-Reciprocal geometric scattering kernel *K_geo_*, derived for a sparse ensemble of surface objects casting shadows on a Lambertian background. It is given by

(11) $$P_{2}=\alpha C\rho \pi r^{2}$$

Here, $\rho =\frac{n}{A}$ is the number density of objects, *n* is the number of objects, and *A* is the average area of the objects. The crowns are assumed to be spheroids with vertical length of *2b*, horizontal width *2r*, and distance *h* to their centers above the ground.

Parameter *P*_3_ is the coefficient for the Ross-Thick volumetric scattering kernel *K_vol_*. It assumes the leaf canopy is dense.

(12) $$P_{3}=\left(1-\alpha \right)\frac{4s}{3\pi }\left(1-e^{-LAI\;Q}\right)$$

The two constants, dimensionless crown relative height (*P*_4_ = h/b), and shape (*P*_5_ = b/r) parameters have been empirically obtained. They are equal to 2 and 1, respectively, in the validated Li-Sparse-Reciprocal Kernel.

### 2.2. Simulation of Land Surface BRDFs Using Ross-Li Model

**Figure 1:**
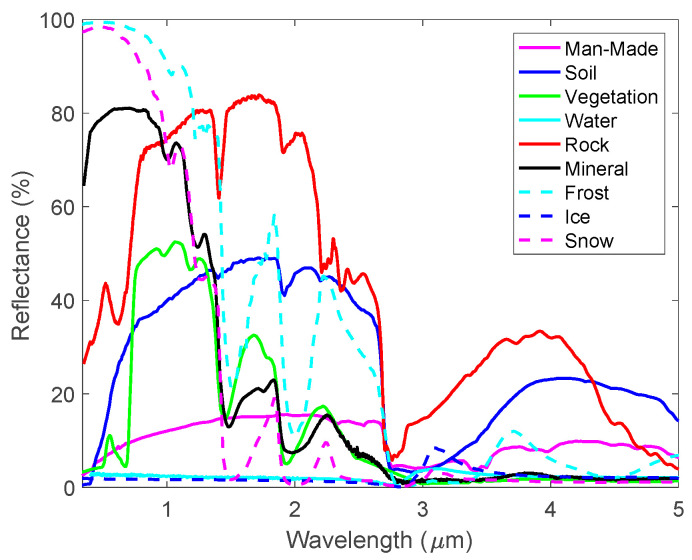
The typical reflective spectra for various land surface materials from the ASTER Spectral Library. They were used to build the land surface bidirectional reflectance distribution functions (BRDFs).

The surface of a specific area of land may consist of bare soils, grasses, trees, and artificial roads and buildings. To simulate the BRDF of a land surface using the Ross-Li model, one has to know the reflectances of these materials. In this work, we adopted the reflectance spectra from the ASTER Spectral Library [37]. These spectra were obtained by measuring the directional hemispherical reflectances alone or by measuring both the directional hemispherical and the bidirectional reflectances of the materials. In the spectral regions of our interest, the spectral library includes the reflectances for 41 different soil surfaces, 24 artificial roofing materials, concretes, road asphalts, and tar. It also includes spectra for grasses (dry and green), trees (deciduous and conifer), snows (fine, medium, and coarse granular), ice, frost, and water. Figure 1 gives examples of the reflectance spectra of these materials in the wavelength region from 200 nm to 5 μm. We selected a total of 892 reflectance spectra from the ASTER library for this study, and those spectra which do not cover wavelengths range from 2.5 μm to 5.0 μm were not used. As the ASTER spectra were measured at different wavelength grids, we interpolated the 892 spectra into a uniform spectral grid of 977 points with spectral range from 200 nm to 5 μm. The spectral dependency of the *C*, *s*, and *R0* parameters in the Ross-Li model are represented by of the ASTER reflectance spectra.

Besides the reflectances of the surface materials, one also needs to know the percentage of the surface area, which is covered by geometric scattering material, the leaf area index (LAI), and the number density of the scattering objects. The geometric scattering area used in this work was randomly varied from 0 to 100%. The LAI is a dimensionless quantity that characterizes plant canopies. It is defined as the one-sided green leaf area per unit ground surface area in broadleaf canopies. Though the LAI value is larger than 1 for the Ross-Li model, it was randomly selected from 0 (bare ground) to up to 10 (dense conifer forests) in this work to include even more surface scenarios. The number density of the scattering objects was in the range of 0 to 0.5.

**Figure 2:**
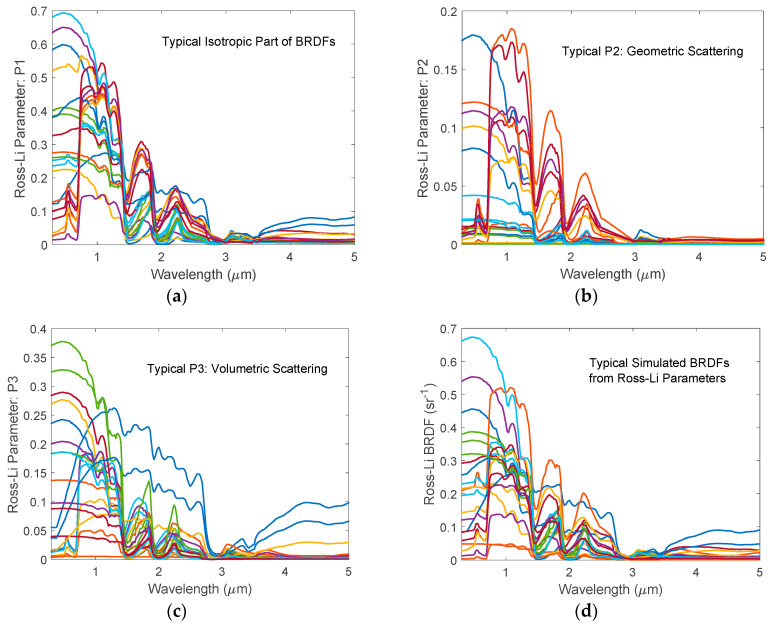
Typical Ross-Li parameters selected from 20,000 simulated samples. (**a**) Isotropic parts of the BRDFs; (**b**) Coefficients of the Li-Sparse-Reciprocal geometric scattering kernel; (**c**) Coefficient for the Ross-Thick volumetric scattering kernel; (**d**) Typical BRDF spectra of the land surfaces obtained using Ross-Li BRDF parameters.

The Ross-Li parameters *P1*, *P2*, and *P3* were calculated using the ASTER reflectance spectra and various parameter values described above. A total of 40,000 BRDF spectra at different satellite zenith angle (0–75°), solar zenith angles (0–85°), and relative azimuth angle (0–180°) were calculated using Equation (1) at the 977 wavelength grids from 0.2 μm to 5 μm. Figure 2 shows typical Ross-Li parameters and the corresponding BRDF spectra used in the BRDF model. It is worth noting that *P1*, which is the isotropic scattering contribution to BRDF, is smaller than 1. This is what one may expect as it is the bidirectional reflectance at *θ_r_* = 0 and *θ_i_* = 0. On the other hand, BRDF may be larger than 1 in some wavelength range for some cases. It is well known that the value of BRDF is not limited to the range of 0 to 1 as it is defined as the ratio of the reflected radiance to the incident irradiance. The unit of BRDF is *sr*^−1^ (per steradian) and its value may be any positive number [48]. The various reflectances, which are defined as the ratio of the reflected flux in the reflective solid angle to the incident flux in the incident solid angle, are dimensionless parameters and are always smaller than 1 due to energy conservation.

The spectral shape of a BRDF is governed by three parts: The shapes of the isotropic (*P1*), the geometric (*P2*), and the volumetric (*P3*) scatterings. The weights of the three parts are determined by the solar and viewing angles as given in the scattering kernels. Using the measured reflectance values for various soils, vegetations, snow, ice, water, roofs, roads, and other artificial materials, one can simulate the hyperspectral BRDF spectra for numerous diverse land surfaces. The simulated BRDFs can be used for many remote sensing applications. In this work, we use a PCA regression technique to relate the Ross-Li BRDF parameters or reflectance values at few preselected wavelengths to the hyperspectral BRDF spectra or reflectance spectra in the wavelength range from 0.2 μm to 5.0 μm. By doing so, we can use the Level 2 BRDF product from multiband satellite instruments such as VIIRS and MODIS to generate HSBR with a large spectral coverage. One can use this HSBR as a first guess for a retrieval algorithm. It can also be used as the surface BRDF input for a radiative transfer model when performing an Observing System Simulation Experiments (OSSEs) simulation for a hyperspectral satellite instrument. Furthermore, the diverse HSBR database can be used to construct a priori error covariance matrix and be used to constrain the shape of the retrieved BRDF for a physical inversion algorithm.

### 2.3. Representativeness of the Simulated BRDFs

**Figure 3:**
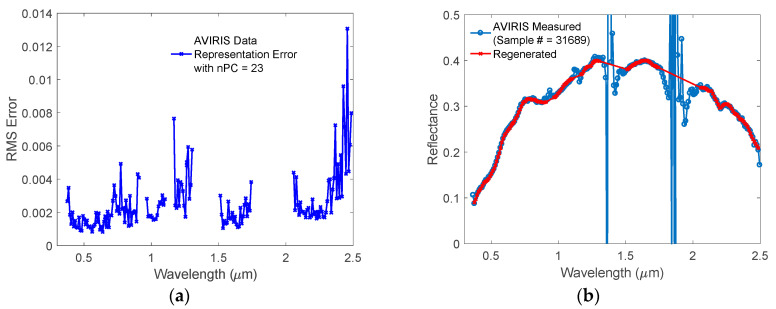
Representativeness of the simulated BRDFs validated using AVIRIS data. (**a**) Root mean square (RMS) error of the regenerated AVIRIS reflectances from a linearly combination of 23 PCs obtained from the simulated training data; (**b**) Randomly selected AVIRIS measured reflectance spectrum vs. the regenerated spectrum.

The simulated land surface reflectance spectra described in Section 2.2 were used to perform the principal component analysis. The resulting Principal Components (PCs) can efficiently capture the BRDR spectral correlations across a wide spectral range. This spectral correlation can be used to generate regression relationships between BRDFs at a few selected wavelengths from a multiband instrument to the hyperspectral BRDFs over a wide spectral range. The diversity of the training data is very important as these BRDFs are supposed to represent all types of land surfaces. To test the representativeness of the simulated BRDFs, we first performed principal component analysis to the HSBR data and concluded that 23 PCs were enough to reconstruct all the HSBR spectra with a root mean square (RMS) error smaller than 0.1% in the whole spectral range. Secondly, we selected 40,000 reflective spectra from the AVIRIS database; these spectra were obtained for various surfaces, such as mountains, forests, sands, lakes, fields, roads, urbans, and suburbs. The 40,000 spectra served as the independent data to test the representativeness of the simulated BRDFs. They were projected onto the 23 PCs to obtain projection coefficients. The PCA regenerated AVIRIS reflectance spectra were obtained by linearly combining the 23 PCs with corresponding projection coefficients. To increase the accuracy, very noisy data in the strong water vapor absorption spectral bands and near the edges of the AVIRIS spectral response range were removed. Therefore, one may consider the remaining measured data as the true values of the reflectance of the land surfaces. Taking the measured AVIRIS data as the true values, the RMS errors of the PCA regenerated data were calculated. The results are shown in Figure 3a. The RMS error of the regenerated reflectance is normally smaller than 0.005, it becomes slightly larger in the long wavelength range. The corresponding relative root mean squares error (RRMSE) is ~1% except those at the noisy edge pixels. The small RMS errors indicate that using 23 PCs generated from our BRDF database can represent the reflectances of the 40,000 AVIRIS measured land surfaces very well. This conclusion is also supported by the comparison of the randomly selected sample from the 40,000 spectra, as shown in Figure 3b. At the shortest and longest wavelengths, the regenerated reflectance values show big difference due to the larger measurement errors in the AVIRIS data. In the spectral region of four strong water absorption bands (0.9 μm, 1.2 μm, 1.4 μm and 1.8 μm), the AVIRIS measured reflectance values are also very noisy due to small signals at the sensor level and potential errors introduced by the atmospheric correction process. At all other wavelengths, the regenerated reflectance overlaps with the original measured one very well, indicating the simulated HSBR database has a very good representation of land surface reflectance spectra.

## 3. Methodology of HSBR Algorithm

Our previous works show that one can use radiances at a few preselected wavelengths to generate the whole radiance spectrum for a hyperspectral sensor [52,53,54]. The principal component-based method also works for the BRDF spectrum [44,45]. One can pick the BRDFs at few representative wavelengths using principal component analysis (PCA) and the method used for our principal component-based radiative transfer model to calculate the hyperspectral BRDFs with very high accuracy. As the multispectral reflectances measured by currently available instruments have fixed surface channel wavelengths, we are not going to select an optimal set of hinge wavelengths in this work. In fact, we use the MODIS available BRDFs to develop our HSBR model directly. The current model will be limited by this wavelength constraint. It is worth to notice that a more accurate model can be obtained if the BRDFs at the optimized wavelengths are available.

According to PCA, the mean removed BRDFs of the land surface may be given by the linear combination of their PCs,

(13) $$B_{nch\times 1}=U_{nch\times npc}Y_{npc\times 1}$$

where *B* is the BRDF, *U* is the matrix of the selected PCs, *Y* is a vector containing PC scores or PC projection coefficients, and *nch* is the total number of channels of the hyperspectral reflectance or BRDF. The *U* matrix is obtained by a singular value decomposition of the covariance matrix of the training hyperspectral BRDF data. The *Y* vector can be obtained by projecting the *B* spectrum onto the *npc* leading PCs $(U_{nch\times npc}).$ The BRDFs in the training database can be easily regenerated using Equation (13) as *U* and *Y* are known from PC analysis with extremely high accuracy.

To calculate the BRDFs of a land surface when hyperspectral data is not available, the PC coefficient vectors *Y*, which correspond to the new land surface, have to be obtained in other ways. The *U* matrix, which captures the spectral variability of the simulated BRDFs, remains the same in the BRDF calculation for the new land surfaces. In previous studies [45,46,47], the PC coefficients were calculated using the PCs and the BRDFs at the satellite/airborne multi-band instrument available wavelengths,

(14) $$C_{npc\times 1}={\left(U_{npc\times nch0}U_{npc\times nch0}^{T}\right)}^{-1}U_{npc\times nch0}B_{nch0\times 1}^{hinge}$$

Here, *nch*0 is the number of channels available for the multiband sensor, which is much smaller than *nch*. For MODIS visible and near infrared surface bands, the *nch*0 equals to 7. The available satellite surface channels are used as the hinge wavelengths in the PCA regression method. The $B_{nch0\times 1}^{hinge}$ is a vector containing the BRDF or reflectance values measured by the multiband satellite instruments such as MODIS. The resulting *C* coefficients are then used to generate hyperspectral BRDF or reflectance spectrum according to Equation (15).

(15) $$B_{nch\times 1}=U_{nch\times npc}C_{npc\times 1}$$

However, the *C* coefficients obtained via Equation (14) are not real PC scores (i.e., *Y* in Equation (13)), which explains why the regression results became unstable when a larger number of PCs were used in the previous studies [45,46]. To solve for the PC scores in Equation (13) with limited *nch*0 wavelengths provided by multiband satellite sensors, we directly relate the needed hyperspectral BRDFs to the satellite measured multiple hinge BRDFs according to Equation (16),

(16) $$B_{nch\times 1}=U_{nch\times npc}D_{npc\times nch0}B_{nch0\times 1}^{hinge}$$

with

(17) $$D_{npc\times nch0}=U_{nch\times npc}^{T}B_{nch\times ns}B_{nch0\times ns}^{T}{\left(B_{nch0\times ns}B_{nch0\times ns}^{T}\right)}^{-1}$$

where the superscript *T* represents transpose of a matrix, and *ns* is the number of HSR spectra used in the training process. The parameter *D* is a coefficient matrix which transforms the multiband BRDFs of the interested land surface, $B_{nch0\times 1}^{hinge}$, to the corresponding PC score vector *Y*. Obviously, the PC scores obtained in our model are not only determined by the hinge point BRDFs or reflectances of the interested land surface, but also by the PCs and BRDFs or reflectances at ALL of the high resolution HSBR wavelengths of the training data.

Equation (16) can be simplified as

(18) $$B_{nch\times 1}=A_{nch\times nch0}B_{nch0\times 1}^{hinge}$$

with

(19) $$A_{nch\times nch0}=U_{nch\times npc}U_{nch\times npc}^{T}B_{nch\times ns}B_{nch0\times ns}^{T}{\left(B_{nch0\times ns}B_{nch0\times ns}^{T}\right)}^{-1}$$

The regression coefficient matrix, *A*, can be precalculated using the same training data as those used in the PCA. It directly transforms the BRDF or reflectance values measured by multiband instruments into HSBR.

**Figure 4:**
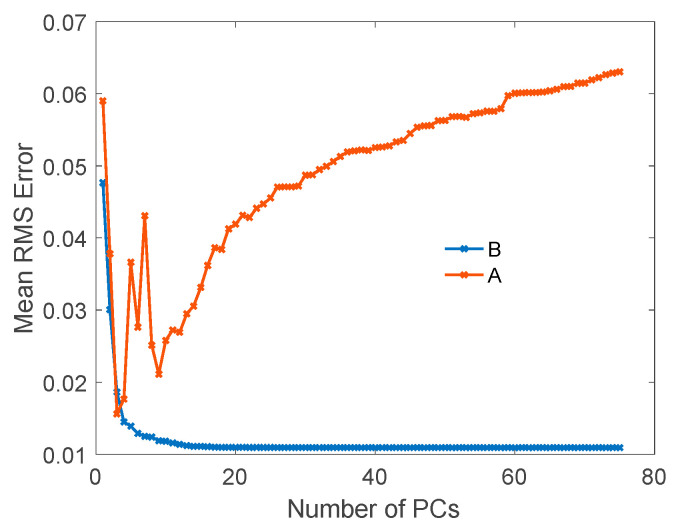
The mean RMS errors of the simulated BRDFs using previous principal component analysis (PCA) regression method (**A**) and our HSBR model (**B**), respectively.

To compare the results calculated using the current HSBR model and the previous model described in references [45,46,47], we calculated the spectral mean RMS error as a function of the PC numbers. The results are shown in Figure 4. The unstable behavior is clearly shown in the previous model and the RMS error becomes even larger with increased number of PCs, as shown in curve A in the plot. On the other hand, as presented by curve B, the mean RMS error in our HSBR model monotonically decreases with the number of PCs.

**Figure 5:**
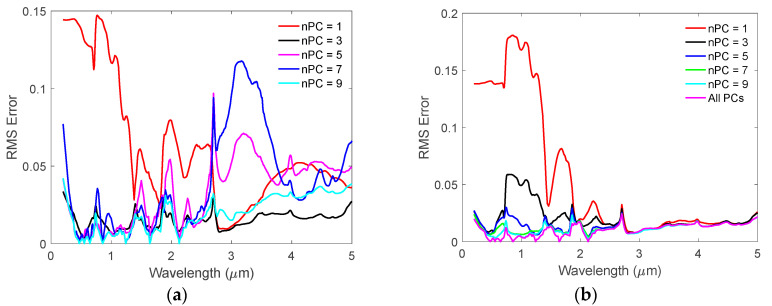
(**a**) The RMS errors of the simulated BRDFs using method described in [43,45]; (**b**) The RMS errors of the simulated BRDFs using HSBR model reported in this paper.

The RMS errors of the simulated BRDFs at different wavelengths relative to the original BRDFs using different numbers of PCs are shown in Figure 5. In Figure 5a, the RMS errors do not decrease monotonically with the increased PC numbers when using the method reported in [43,45]. This was also reported in their previous work [46]. On the other hand, the RMS errors obtained using our HSBR model decrease with the increase in the number of the principal components, *nPC*. This is what one may expect from the PCA theory. The “all-PC” result shown in Figure 5b is obtained by using Equations (18) and (19) with all PCs included. It has the smallest RMS error as expected. The change in RMS error becomes negligible when the number of PCs is larger than 20. Thus, we will only show the results in the following parts of the paper using 20 PCs.

## 4. Model Validation

### 4.1. Validation of the HSBR Model Using Simulated BRDFs

**Figure 6:**
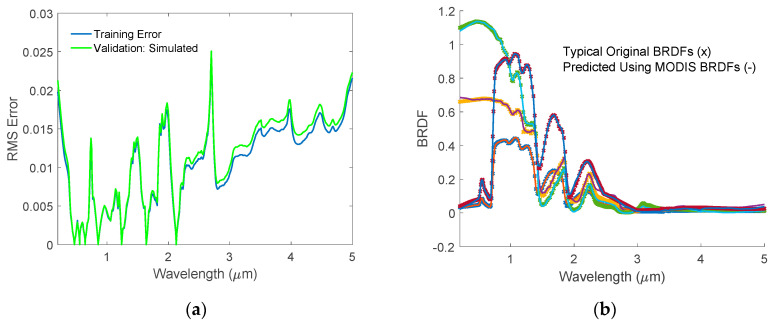
(**a**) Validation RMS errors for the hyperspectral BRDF spectra. Blue line is for training error while the green line represents validation error using the independent simulated BRDFs; (**b**) Validation results for BRDF spectra. The symbol ‘x’ indicates the original data while the symbol ‘-‘ is for the simulated results. The simulated results overlap with the original ones very well.

Utilizing the pre-saved PCs and the *A* matrix, one can simulate the high-resolution hyperspectral BRDF or reflectance using multiple BRDFs or reflectances measured by airborne and satellite sensors. If the MODIS product is used, one can use the seven MODIS BRDFs or reflectances to simulate the hyperspectral BRDF or reflectance with the help of the pre-saved matrices. To validate the HSBR model using simulated BRDFs, we simulated 18,131 independent BRDF spectra, which were not used in the PC analysis process. These BRDFs hyperspectra were regenerated using seven BRDFs values at the MODIS surface channel wavelengths and the pre-saved *A* matrix of our HSBR model. The RMS errors of the regenerated BRDFs were calculated and shown in Figure 6. The RMS errors are usually smaller than 0.02 and the RRMSEs are ~2% in the wavelength range from 0.5 to 1.25 μm. The RRMSEs increase in other wavelength ranges due to both the small BRDF values and the lack of hinge points in these spectral regions. Figure 6b shows some selected cases from the independent validation dataset. Each of them has very different spectral features, which implies that they are for different land surface scenarios. In all of the cases, the HSBR model regenerated BRDF spectrum agrees very well with the original BRDF spectrum, indicating that excellent training results were obtained for the HSBR model.

### 4.2. Validate HSBR Model Using USGS Vegetation Database

Although the HSBR model is validated with excellent results by the independent simulated BRDFs, it is critical to check if it works for real measured reflective spectra. Thus, we will use both USGS and AVIRIS databases to validate the HSBR model.

**Figure 7:**
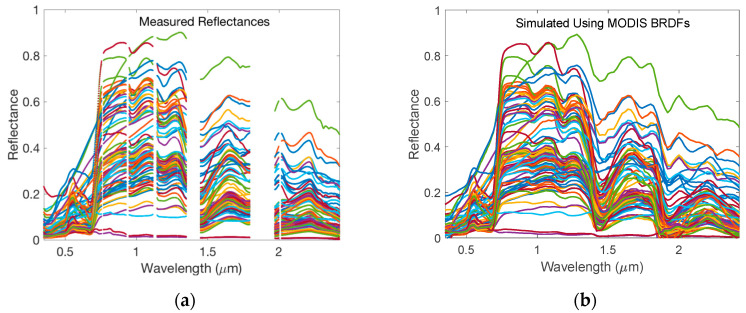
(**a**) The measured reflectances for vegetation from USGS database. The white space areas are wavelength ranges in which the reflectances cannot be measured accurately for some samples; (**b**) The simulated reflectances using HSBR model. The reflectances at MODIS wavelengths used in HSBR model were extracted from the USGS database.

The USGS vegetation database includes spectra for biological materials: Plant components such as leaves, flowers, and barks. It also includes vegetated areas having more than one species present, lichens, biological soil crusts, and mixtures with vegetation [36]. The selected spectra were measured using Analytical Spectral Devices (ASD) spectrometers with wavelength range from 0.35 μm to 2.5 μm. The measured spectra have 2151 channels (bands) and some of the channels were assigned as bad bands by a value of −1.23 × 10^34^. These bad bands are in the wavelength range of 0.350 to 0.413 μm, 0.756 to 0.770 μm, 0.928 to 0.950 μm, 1.116 to 1.146 μm, 1.350 to 1.450 μm, 1.795 to 2.019 μm, and 2.425 to 2.500 μm, respectively. They are located in the white space areas in Figure 7a.

To validate our HSBR model using the independently measured vegetation spectra, we first interpolated the measured spectra to the 7 MODIS wavelengths, and then simulated the whole HSBR spectra from 0.2 to 2.5 μm using the resulting reflectances at the seven MODIS wavelengths. The results are shown in Figure 7b.

As the PCs derived from our HSBR database cover the whole spectral range from 0.2 to 2.5 μm, the reflectance values at those bad channels can be easily obtained using our HSBR model. Once the PC scores are generated using the multiband information (or other good spectral channels in the USGS hyperspectral reflectance data), the resulting hyperspectral reflectance should have continuous values within the 0.2 to 2.5 μm spectral range. This provides a great way to achieve a reliable reflectance of the land surface at wavelengths which cannot be measured accurately due to strong atmospheric water absorptions.

**Figure 8:**
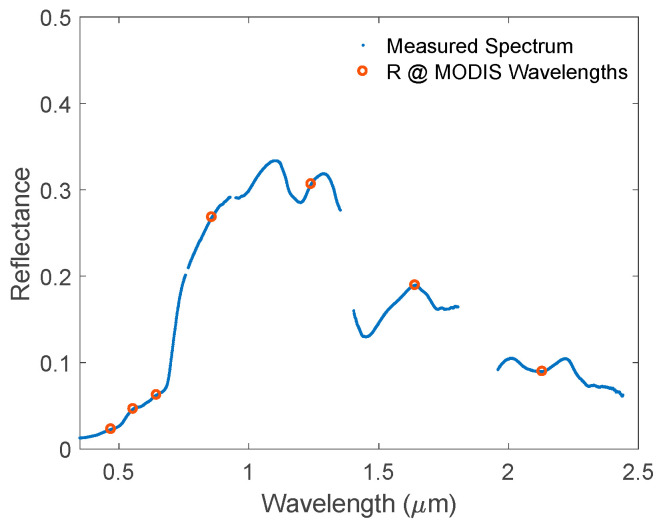
A randomly chosen experimental reflective spectrum from USGS database (blue). The red circle indicates the reflectances at the MODIS available wavelengths.

Though there are always errors in measured data, the seven MODIS surface channel wavelengths are not close to the bad bands (i.e., strong water absorption regions), as shown in Figure 8. Using these reflectances and the HSBR model, one can easily calculate the reflective spectrum from 0.2 to 2.5 μm.

**Figure 9:**
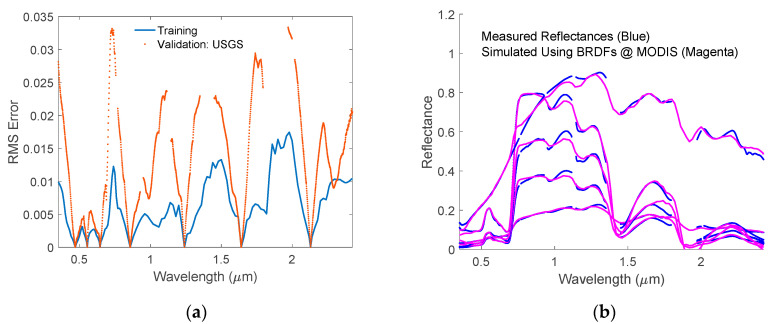
(**a**) The RMS errors of simulated spectra using HSBR model with respect to experimental measurements in the USGS vegetation database; (**b**) Randomly picked experimental reflective spectra from USGS vegetation database (-) and the corresponding simulated spectra obtained using HSBR model (--).

To check how well the HSBR model-generated spectra agree with the experimental measurements, we calculated the RMS errors between the original USGS reflectance spectra and those generated using the 7 MODIS surface channel reflectance values. The results are shown in Figure 9a. The RMS errors, which are smaller than 0.035, are acceptable for remote sensing applications. It is reported that the differences in the measured reflectances of the same sample using different equipment may be as large as 0.05 in some wavelength ranges [36]. It is also worth to note that the maxima of the RMS errors appear at wavelengths close to the edge of bad bands (due to low transmittance through the Earth’s atmosphere). Therefore, the relatively large differences in these wavelength areas between the simulated reflectances and the measured ones are probably not due to the simulation accuracy. It is more likely due to the relatively large experimental errors caused by both strong atmospheric absorptions and procedure used to extract surface reflectances from radiance data.

Figure 9a shows the differences between the simulated spectra and the measured ones are small enough for actual applications. To further examine the accuracy of this method, we selected five reflectance spectra with reflectance values at 1.084 μm to be 0.2, 0.4, 0.6, 0.8, and 1.0, respectively. Figure 9b shows the five experimental spectra and their corresponding simulated spectra using 7 MODIS surface channel reflectance values. It is obvious that the simulated reflectance spectra agree well with the measured ones. One can safely use the simulated reflectances with HSBR model to any land surface albedo related applications.

### 4.3. Validate HSBR Model Using AVIRIS Database

To further validate the HSBR model, we selected one of the AVIRIS data cubes named f140530t01p00r16_refl. It was measured on 30 May 2014 at UTC 17:47 with solar elevation angle of 62.08 degree and solar azimuth angle of 112.95 degree. We chose this data cube because it covers mountains, forests, sands, roads, urban, and suburb areas. The diversity of these data provides a good test to the applicability and accuracy of the HSBR model.

**Figure 10:**
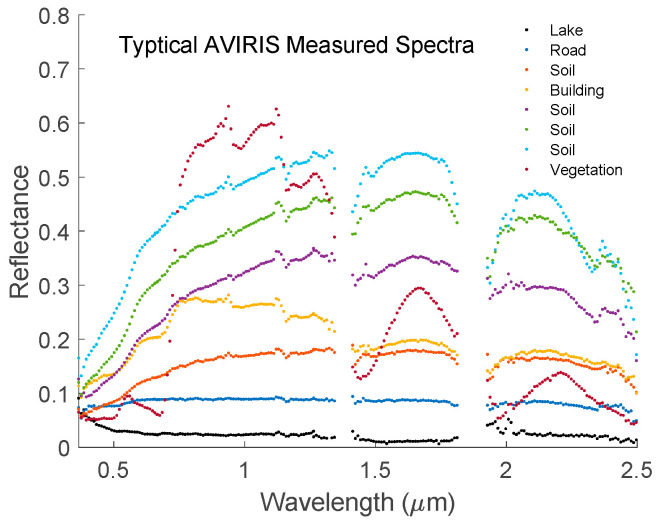
Typical measured reflectances by the AVIRIS instrument. The blank areas are wavelength ranges in which the reflectances cannot be measured accurately due to strong water absorption.

The AVIRIS hyperspectral cube has a dimension of 3337 × 850 × 224. The 1st and 2nd dimensions are for spatial coordinates while the 3rd one is spectral coordinate. Among these data, 36,476 land surfaces were selected by picking every 8th scans from the first line for the first dimension while picking every 8th pixels from the first pixel for the second dimension of the data cube. Figure 10 shows typical measured reflectances. Due to strong water absorptions, the measured data are very noisy in the wavelength range of 1.353 to 1.412 μm and 1.821 to 1.927 μm respectively.

**Figure 11:**
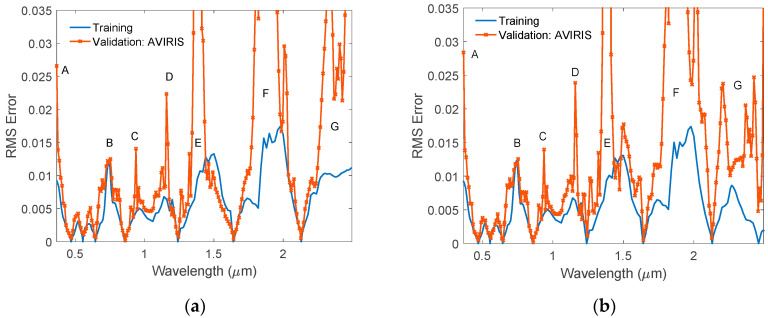
(**a**) Validation RMS errors of the simulated reflectances using HSBR model. The measured AVIRIS data were assumed to be the “true values”; (**b**) Validation RMS errors of the simulated reflectances using HSBR model with 8 hinge wavelengths. The additional hinge point is at 2.46 μm. The measured AVIRIS data were assumed to be the “true values”.

Following the same procedure as we discussed in the USGS vegetation case, we simulated the AVIRIS data cube using reflectance values at the seven MODIS surface band wavelengths. The reflectances at the MODIS wavelengths were obtained by interpolating the measured spectra as we did in the USGS case. The RMS errors between the AVIRIS measured and our HSRB model generated reflectance spectra are given in Figure 11a. The RMS errors are larger than 0.05 near the two strong water absorption bands (marked as E and F). The RMS errors at the shortest wavelength (marked as A) and the longest wavelength are also big because of the large measurement noises at the edge pixels. Other channels with RMS errors greater than 0.01 are approximately 0.75 μm (B), 0.94 μm (C), 1.16 μm (D), and in region beyond 2.25 μm. The error around 0.75 μm may be caused by the atmospheric correction uncertainty due to the strong absorption of O_2_ and water. Similarly, the errors around 0.94 μm and 1.16 μm are probably due to the large reflectance errors in the measured data in the strong water absorption bands. The error in the long wavelength region (>2.25 μm) may come from simulation error due to the lack of information beyond the longest MODIS wavelength at 2.1 μm. This error will be significantly reduced if information is available at one of the longer wavelength region. To demonstrate this idea, we added an additional hinge point at 2.46 μm and repeated the training and validation processes using eight hinge wavelengths. The results are shown in Figure 11b. Obviously, the RMS error in the long wavelength region (G area) is reduced significantly. In all other spectral regions, the simulated spectra fit the measured ones very well, with RMS errors normally smaller than 0.01. Generally, the AVIRIS reflectance is within several percent of field measured reflectance [55]. Thus, the error in the simulated reflectance using HSBR model is comparable to the reflectance measurement error.

**Figure 12:**
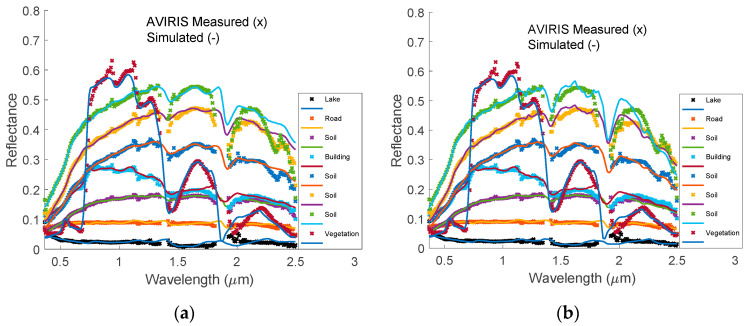
(**a**) Randomly selected reflective spectra measured by AVIRIS instrument (x) and the corresponding simulated spectra (-) using HSBR model with 7 hinge wavelengths. The big differences occur at water absorption bands and in region with wavelength larger than 2.25 μm; (**b**) Selected reflective spectra measured by AVIRIS instrument (x) as shown in (**a**) and the corresponding simulated spectra (-) using HSBR model with 8 hinge wavelengths. The fit in long wavelength range is greatly improved. The noisy AVIRIS measured reflectances in the region from 1.353 μm to 1.412 μm and region from 1.821 μm to 1.927 μm are not shown in these plots. By using 8 or more hinge wavelengths in our HSRB model, we can fill these spectral gaps using spectral correlation information contained in the HSRB principal components.

Figure 12a shows some randomly selected AVIRIS experimental spectra with their corresponding simulated spectra. In general, the simulated spectra fit the measured ones very well. The main differences are located in the strong absorption bands, which have large measurement error and large atmospheric correction errors. Figure 12b shows the same measured spectra and the simulated ones using eight hinge wavelengths. The quality of the simulated spectra in the long wavelength range is greatly improved due to the added information at 2.46 μm.

It was reported that AVIRIS-derived surface reflectance spectra have larger noises compared to laboratory measured spectra due to complicated atmospheric conditions [56]. Gao et al. developed an algorithm to retrieve the surface reflectance by modeling the absorption due to water vapor, carbon dioxide, ozone, nitrous oxide, carbon monoxide, methane, and oxygen [57]. However, the AVIRIS-derived reflectances are still very noisy in the water vapor absorption bands, as shown above. The quality of the AVIRIS reflectance product may be further improved significantly in the water absorption regions if our PCA derived HSBR is used as a priori constraint in a physical retrieval algorithm.

We also used millions of other AVIRIS data measured on other days and other locations at different solar elevation angles to validate our model. One of the measurements was carried out on 20 October 2015 at a solar elevation angle of 37.73 degree at the site named Yosemite-NEON Box 10. Over 12 million spectra were obtained in this flight. Another measurement was done on 13 October 2015 at a solar elevation angle of 42.9 degree at the site marked Tahoe Box Line 1. Over 8 million hyperspectral reflectance spectra were acquired in that flight. We used the hinge reflectances at the seven MODIS available wavelengths to regenerate their corresponding hyperspectral reflectances. Similar results were obtained as we reported for the measurements given in file f140530t01p00r16_refl. These results strongly indicate that our model is applicable for different land surfaces.

## 5. Conclusions

The hyperspectral bidirectional reflectance (HSBR) model we developed in this work has been successfully used to simulate various land surface reflective spectra using BRDF parameters or multispectral reflectance at preselected wavelengths. The simulated spectra using BRDFs or reflectances at MODIS surface channel wavelengths for various land surfaces agree with the experimental measurements very well. In spectral regions which are away from the channels that are affected by atmospheric molecular absorption and scattering, the standard deviations between the simulated spectra and remote sensor measured reflectance spectra are normally smaller than 0.01, which are comparable to the experimental errors. This indicates that the HSBR model is an accurate and powerful tool to simulate the land surface BRDFs for remote sensing applications.

As our BRDF database is derived from the ASTER reflectance spectra and the Ross-Li BRDF model, it provides high-quality reflectance spectra with continuous spectral coverage from 0.2 to 5.0 μm, including spectral regions with strong atmospheric gas absorptions, such as the water bands at around 0.9 μm, 1.2 μm, 1.4 μm and 1.8 μm. On the other hand, the surface reflectance spectra derived from airborne or satellite sensors usually have large errors for those wavelengths with strong atmospheric absorptions. The strong gas absorption may obscure the surface information, leading to the noisy channels in the reflectance product even after atmospheric correction. One can use satellite or airborne sensor-measured reflectance values at a few selected atmospheric window spectral regions and our HSBR model to produce high quality BRDFs for these noisy channels. The BRDF model developed in this study can be used to generate first guesses for a retrieval algorithm for satellite or airborne remote sensing instruments. The BRDF PCs and associated statistics can be very useful in generating background and a priori constraints for a satellite retrieval algorithm.

The HSBR model can also be used for intercalibrations of satellite instruments [12,13,58]. Performing satellite instrument intercalibrations is critical for understanding climate processes, environment changes, and weather forecasting. The hyperspectral Climate Absolute Radiance and Refractivity Observatory (CLARREO) instrument is proposed to serve as a community standard for intercalibration [59]. To use the CLARREO pathfinder instrument, which will be launched onboard of international space station in 2023 timeframe, as a reference standard to calibrate current or future instruments, such as the broad band CERES instrument, one needs to match the spectral coverage of the two instruments first. It is very important to know the reflectance values at wavelengths that cover both the CLARREO and CERES wavelengths. Unfortunately, no hyperspectral reflectance product is available for these satellite instruments. The HSBR model can be used to solve this problem. The HSBR model will be a powerful tool for all land surface related remote sensing applications.
